# A Review of Emerging Tear Proteomics Research on the Ocular Surface in Ocular Allergy

**DOI:** 10.3390/biology11020312

**Published:** 2022-02-16

**Authors:** Esrin Aydin, Poshmaal Dhar, Moneisha Gokhale, Luke Chong, Serap Azizoglu, Cenk Suphioglu

**Affiliations:** 1 School of Life and Environmental Sciences, Deakin University, Waurn Ponds, VIC 3216, Australia; eaydi@deakin.edu.au; 2 Deakin Optometry, Deakin University, Waurn Ponds, VIC 3216, Australia; moneisha.gokhale@deakin.edu.au (M.G.); luke.chong@deakin.edu.au (L.C.); serap.azizoglu@deakin.edu.au (S.A.); 3 School of Medicine, Deakin University, Waurn Ponds, VIC 3216, Australia; posh.dhar@deakin.edu.au

**Keywords:** ocular allergy, allergic conjunctivitis, keratoconus, proteome, biomarker

## Abstract

**Simple Summary:**

Ocular allergy is a localised form of allergy occurring on the surface of the eye and surrounding tissues. Typical signs and symptoms of ocular allergy include itching, redness, swelling of the eyelids and inflammation. Emerging studies on ocular allergy have shown that tears collected from ocular allergy sufferers show significantly different protein contents than in healthy populations. Differences in protein contents in tear samples have been hypothesised to be caused by a number of allergy-mediated factors, including long-term inflammation and eye-rubbing. Excessive eye-rubbing due to allergy-associated itch has also been shown to have significant effects on the physical shape of the eye, thereby potentially causing progressive vision problems in ocular allergy sufferers. This review aims to summarise and explore recent findings in ocular allergy protein research. This is to help determine which ocular surface proteins differ between ocular allergy sufferers and healthy controls, and the role each protein may play in the underlying chemistry of ocular allergy. Additionally, potential benefits of expanding the current pool of research into ocular surface proteins in ocular allergy sufferers in terms of diagnosis and treatment of the condition is discussed.

**Abstract:**

Ocular allergy is an immunoglobulin E-mediated Type I hypersensitivity reaction localised to the ocular surface and surrounding tissues. Primary signs and symptoms of ocular allergy include itching, redness, irritation and inflammation. Eye-rubbing caused by itching has been shown to alter ocular surface protein concentrations in conditions linked to ocular allergy such as keratoconus. In keratoconus, the cornea begins to thin and sag over time, leading to progressive vision loss and blindness in severe conditions. Due to the high incidence of ocular allergy sufferers rubbing their eyes in response to symptoms of itching, the protein landscape of the ocular surface may be significantly altered. Differential protein expression caused by long-term inflammation and eye-rubbing may lead to subsequent changes in ocular surface structure and function over time. This review aims to summarise and explore the findings of current ocular allergy proteome research conducted using techniques such as gel electrophoresis, mass spectrometry and lab-on-a-chip proteomics. Proteins of interest for this review include differentially expressed immunoglobulins, mucins, functional proteins, enzymes and proteins with previously uncharacterised roles in ocular allergy. Additionally, potential applications of this research are addressed in terms of diagnostics, drug development and future research prospects.

## 1. Ocular Allergy Overview

**Figure 1:**
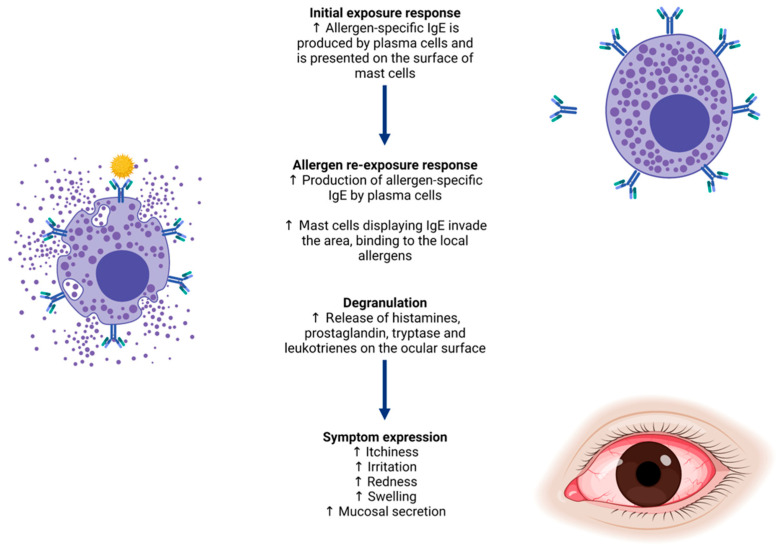
Allergy biopathway from immunoglobulin E (IgE) production for a specific allergen, to signs and symptoms of ocular allergy experienced upon re-exposure. These include itchiness, redness, swelling, irritation and inflammation of the ocular surface and surrounding tissues.

The current proteomic landscape of the ocular surface in ocular allergy outlines a compelling narrative, potentially linking ocular allergy to increased concentrations of inflammatory and immune defence proteins compared to normal ocular surface conditions. Ocular allergy is a localised subset of immunoglobulin E (IgE)-mediated allergy (Type I hypersensitivity), whereby specific IgE antibodies are raised against allergic molecules from sources such as pollen, animal dander and fungal spores, upon initial exposure [1,2]. When reintroduced to the ocular surface, these allergic molecules trigger a cascade of protein-mediated cellular responses, eventuating in the release of allergy mediators such as histamine, immunoglobulins and enzymes from conjunctival mast cells and eosinophils. Following release, mediators are carried across the ocular surface via tears, triggering common signs and symptoms of ocular allergy (Figure 1), such as inflammation, redness, swelling of the eyelids, irritation and itching [3].

When considering the full scope of signs and symptoms experienced by ocular allergy sufferers, a clear pattern emerges of prolonged itchiness and inflammation due to the allergy-induced release of histamine, leading to excessive eye-rubbing [4]. If experienced mildly and acutely, these symptoms may appear to be manageable and unharmful. The issue occurs when ocular allergy sufferers experience incessant flareups. Downstream effects of sustained inflammatory assault on the ocular surface (due to signs and symptoms of ocular allergy) have not yet been characterised fully and require further research. Signs and symptoms of particular concern include itchiness and inflammation as ocular allergy sufferers may seek relief by excessively rubbing their eyes or by using over-the-counter remedies that are not necessarily best suited for their symptoms. This may perpetuate an indefinite cycle of irritation and inflammation until appropriate treatment is sought. Excessive eye-rubbing due to itching has the potential to become harmful over time, causing changes to the ocular surface structure and protein composition [5]. The collagen matrix of the cornea has been shown to be disrupted by prolonged eye-rubbing, causing semi-permanent damage that may lead to progressive vision loss [5]. These changes are mirrored in a condition called “keratoconus” (corneal ectasia) whereby the cornea thins and sags over time [5]. Recent advances in optometric research have suggested a link between excessive eye-rubbing behaviours (such as those in ocular allergy) and keratoconus in adults [6]. Additional research is urgently needed to investigate the full scope of ocular surface changes resulting from patient self-management strategies for ocular-allergy related symptoms at the biochemical, cellular and tissue levels. 

The impacts of ocular allergy on daily quality of life have been thoroughly researched in recent years. Namely, a study by Stull et al. in 2009 outlined the significance of ocular allergy on daily life [7]. Patients were not given any medications, and were shown to have decreased visual acuity, altered corneal topography, poor sleep quality, impaired productivity and overall decreased mood [7,8]. Corneal thickness was shown to be consistently reduced in ocular allergy sufferers by approximately 4.4 μm when compared to healthy controls as a direct result of eye-rubbing (*p* = 0.001) [8]. Similarly, participants with ocular allergy had a lesser duration and quality of sleep than normal, accompanied by mild photophobia in waking hours due to ocular irritation [7,9]. The study by Stull et al. assessed sleep quality in ocular allergy sufferers using the Medical Outcomes Study Sleep Scale-12 questionnaire, consisting of 12 questions split into assessments of sleep quality, duration, drowsiness and time taken to fall asleep over a week-long period [7]. The *p*-values for relationship between ocular symptoms and poor sleep were significant in two categories pertaining to sleep problems (*p* = 0.21 and 0.20, respectively), sleep disturbance (*p* = 0.19), sleep shortness of breath or with headache (*p* = 0.19) and sleep somnolence (*p* = 0.15) [7]. Additionally, a 2016 study addressing sleep and mood disturbances in patients suffering from ocular disorders found that among 78 ocular allergy sufferers, the mean Hospital Anxiety and Depression Scale score was 8.9 ± 5.3 [9]. Any score over 8 is indicative of symptoms of depression and anxiety, showing clearly that ocular allergy sufferers may experience mood fluctuations due to symptoms [9]. Other factors relating to day-to-day habits and lifestyle, such as ability to play outdoors, swim, socialise, work and exercise, were reportedly also negatively impacted by symptoms of ocular allergy such as itching, redness and irritation [10,11]. In order to reduce the effects of ocular allergy on quality of life for the large number of sufferers globally, it is imperative that accurate diagnostic and treatment protocols are developed urgently [7,12]. An approach utilising the detection of ocular surface biomarkers in human tears associated with ocular allergy sufferers may serve as a viable diagnostic testing method, thereby preventing long-term changes and detrimental outcomes to the cornea and ocular surface in ocular allergy sufferers. Thus, the focus of this review article is the evaluation of the literature on the current proteome landscape of the ocular surface in ocular allergy to identify potential biomarkers or gaps in the knowledge that may pave the way for new and exciting diagnostic and therapeutic research.

## 2. Ocular Surface Biomarkers

Biomarkers are proteomic, genetic or lipidomic characteristics unique to and indicative of a particular biochemical process or pathological pathway [13]. Clinical implementation of biomarker research has been applied in non-ocular surface disorders, such as type 2 diabetes and cystic fibrosis, for the diagnosis and monitoring of disease progression in the past [14,15,16,17]. Ocular surface protein biomarkers for diseases such as dry eye disease, meibomian-gland dysfunction and keratoconus have already been characterised in recent research, however, have not yet been implemented clinically due to a lack of testing protocols appropriate for commercial laboratory screening [18,19,20,21]. Biomarker detection methods may greatly improve the prognosis of ocular surface disorders in patients by allowing for the early detection and implementation of effective management and treatment strategies prior to symptom expression on the ocular surface. Symptom prevention and management strategies are the most effective ways to minimise damage to the ocular surface and prevent downstream effects of itching and eye-rubbing. While not yet clinically utilised, continued research into the relationship between ocular allergy and protein expression on the ocular surface could provide a beneficial insight into unique disease biomarkers. 

Research into lipid and gene biomarkers of ocular allergy has been previously limited due to lack of sufficient analytical techniques, however protein biomarkers have emerged as an exciting and promising new field of research in recent years [14,15,16,17,18,19,20,21]. Protein biomarkers specifically may be detected using a number of assays such as magnetic bead multiplex, automated electrophoretic technology (AET), mass spectrometry (MS) and sodium dodecyl sulphate polyacrylamide gel electrophoresis (SDS-PAGE). The development of rapid, highly accurate testing measures such as these has made it possible for modern research in ocular allergy proteomics to grow, particularly through the formation of more time- and cost-efficient workflows utilising multiple techniques in one analysis. These techniques may also contribute to highly accurate diagnosis of ocular surface disorders by testing basal tear samples [22,23,24].

## 3. Current Tear Collection and Biomarker Analysis Techniques in Ocular Allergy Research

**Figure 2:**
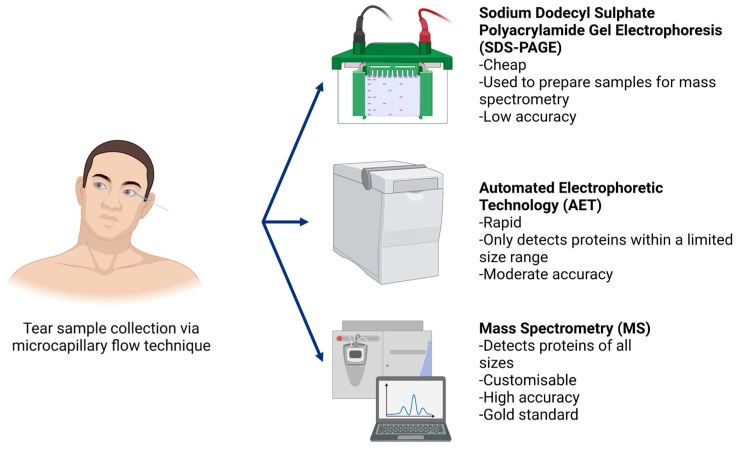
In order to collect human tear samples using the microcapillary flow technique, participants were asked to sit facing forward with their heads tilted all the way to one side to ensure basal tears from the surface of the eye pooled in the lateral canthus (outer corner of the eye). With sterile, gloved hands, glass microcapillary tubes were used to draw tears from the ocular surface. Following sample collection, proteome analysis is typically conducted using sodium dodecyl sulphate polyacrylamide gel electrophoresis (SDS-PAGE), automated electrophoretic technology (AET) or mass spectrometry techniques (in order of increasing accuracy).

Human tears are a compelling sample type as they are a great source of proteins collected directly from the ocular surface using non-invasive techniques [25]. Human tears constantly replenish, gathering regulatory proteins and potential ocular allergy biomarker proteins as they wash over the ocular surface. Proteomic analysis in ocular allergy favours tear collection methods such as microcapillary flow that minimise external sources of itchiness and irritation on the ocular surface. Microcapillary tear collection is simple, does not introduce irritation as a confounding variable, and permits extraction of good quality protein samples (up to 10 μg/μL) [26,27,28]. As shown in Figure 2, in this technique a glass microcapillary tube is placed on the outer corner of the eye, collecting tears normally residing in the inferior fornix via capillary action. Thus, basal tears are collected by capillary action. Proteins are extracted from glass tubes for analysis with minimal effort [27]. In terms of participant comfort, microcapillary flow provides a high level of control over tear volume to be collected while allowing for frequent breaks to blink if necessary. 

Human tear samples gathered by microcapillary collection should be limited to volumes below 60 μL, as greater volumes have been associated with excessive watering, potentially due to introduced ocular stress [28]. Typical tear samples range from 5 to 45 μL [29]. Additionally, a paper by Nakamura, Sotozono and Kinoshita reported undetectable levels of proteins in stimulated tears compared to unstimulated tears due to dilution, as collected using microcapillary flow techniques [28]. It is thereby most effective to use unstimulated tear samples when characterising natural protein environments of the ocular surface both in healthy individuals and in ocular allergy sufferers.

Proteins extracted from human tear samples have traditionally been quantified using a combination of different methods reliant on the goals of the study. Methods such as SDS-PAGE are often coupled with MS to identify protein contents from within trypsin-digested gel segments with high accuracy. The placement of protein bands within a sample can be used to identify protein constituents and show if sample degradation has occurred [30]. SDS-PAGE is able to be used for characterisation of ocular allergy biomarker proteins, however, is not able to quantify total protein concentration and may not show all present constituents, particularly if they are similar in molecular weight [30,31]. 

MS is a far more advanced method, capable of identifying proteins within a sample to increasing specificity and accuracy as high throughput technology develops over time; as a result of these technological advancements, progressively lower sample volumes are required for MS analysis, with some recent publications using volumes as low as 5 μL to identify protein contents of tear samples from healthy participants [32]. 

The specificity and accuracy of MS proteome analysis has greatly increased over the last 16 years. MS analysis of tear samples in subjects with no ocular conditions occurred as early as 2005, with a paper written by Li et al. citing the identification of 54 proteins in the tears of one individual [33]. Just 7 years later, a paper by Zhao et al. had streamlined the protein characterisation process using MS and identified 1543 proteins in a pooled tear sample of four participants collected using Schirmer strips [34]. A much more recent paper published in 2021 used MS techniques to identify 890 proteins from sample tear volumes between 4 and 10 μL to a high level of reproducibility (mean R^2^: 0.81 ± 0.17) [32]. This process involved the use of liquid chromatography–mass spectrometry (LC–MS), which had the added advantage of increased precision and accuracy by separating each constituent before protein identification based upon molecular weight and peak height [32]. This process negated the need for upstream fractionation through techniques such as electrophoresis or SDS-PAGE. Results obtained using LC–MS may be later confirmed through Western blotting analysis [32]. As time progresses and the techniques used for proteome analysis are streamlined, the capacity for more in-depth cataloguing of regularly expressed tear proteins in subjects without ocular conditions increases. Thus, a comparative database may be generated for future ocular allergy research to identify biomarkers that may not be significantly expressed in healthy tears.

The use of LC–MS has also been documented in a 2017 paper on comparative protein analysis between healthy controls, dry eye sufferers and meibomian-gland dysfunction sufferers [35]. This study used LC–MS to conduct initial wide-spectrum proteomic assays to identify potential targets for further analysis [35]. Of the 135 proteins of interest identified, 26 were found to undergo expression changes between experimental groups (dry eye and meibomian-gland dysfunction) compared to healthy controls [35]. Protein quantitation was then carried out using label-free spectral counting (APEX quantitation) based upon LC–MS data. LC–MS therefore provides highly accurate data (99% confidence level) with high reproducibility and low sample volume requirements [35]. LC–MS has not been applied to tear sample proteomics analysis in ocular allergy studies thus far. However, its success in investigating potential biomarkers of dry eye and meibomian-gland dysfunction as well as identifying proteins in healthy human tears indicate that it is a promising technique for use in ocular allergy research.

An emerging “lab-on-a-chip” automated electrophoresis technology (AET) has also recently gained popularity in ocular allergy tear analysis studies despite primarily being used with other sample types such as blood, sputum and buccal cells [23,36,37,38]. The speed, reproducibility and accuracy of AET has thus made it a highly desirable technique for tear analysis [24,39,40,41]. This instrument is able to separate, quantify and characterise protein constituents in tear samples as small as 2 μL [24]. This is particularly advantageous as typical tear samples are between 5 and 45 μL [29]. Accuracy values for reproducibility and repeatability were 0.998 and 0.995, respectively [24]. The “lab-on-a-chip” equipment can be paired with a number of compatible kits to fit the size range for proteins of interest from 5 to 250 kDa, while detecting concentrations between 20 and 2000 ng/μL [24,41]. 

Multiple recent proteomic studies using human tears have carried out analysis with the AET due to the speed, reproducibility and accuracy of this assay [24,39,40,41]. A study on dry eye from 2012 compared AET to a workflow of SDS-PAGE and MS. Findings showed that concentrations of primary tear constituents such as lipocalin, lactoferrin, lysozyme and serum albumin were easily identified in similar quantities by both AET and SDS-PAGE/MS methods [24]. Speed and cost considerations may mean that AET is preferable in situations aiming to detect proteins of a specific size range, however MS and LC–MS are much better suited for wide-spectrum proteomic approaches in exploratory ocular allergy research. Studies focusing on proteomic biomarkers of ocular allergy may be a promising direction for future research, as ocular surface proteomes may differ between healthy controls and ocular allergy sufferers.

## 4. Protein Biosignatures in Ocular Allergy

**Figure 3:**
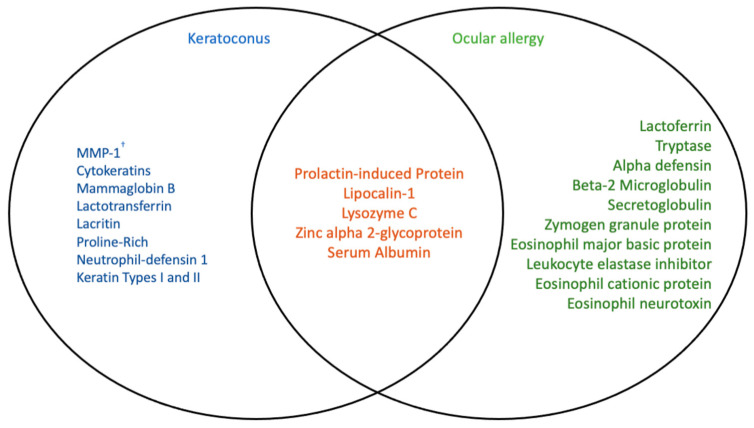
Comparison of differentially expressed proteins in patients with keratoconus and ocular allergy, and proteins that are altered in either condition [3,21,40,47,48,50,51,52,53,54,55,56,57]. Ocular allergy and keratoconus have been linked in recent research through ocular surface changes caused by increased eye-rubbing experienced by sufferers of both conditions. Protein expression on the ocular surface in both conditions has been studied recently, as overlapping key proteins appear to be affected similarly. ^†^ MMP—matrix metalloproteinase.

A number of tear proteome studies on keratoconus patients have uncovered a pattern of irregular protein expression occurring on the ocular surface. Proteins of altered expression in keratoconus may have roles in surface tension stabilisation, pro-secretion and mitogenesis, or antimicrobial activity or be merely involved in cellular infrastructure [42,43,44,45]. More importantly, some properties of these proteins include corneal collagen degradation, vascularisation, wound healing and inflammation, potentially due to excessive eye-rubbing [18,46,47,48]. Excessive eye-rubbing has been well documented, likely causing mild ocular surface trauma that can lead to further irritation and inflammation [49]. In order to return to homeostasis succeeding this, the eye must release high concentrations of anti-inflammatory and wound-healing proteins [48]. As shown in Figure 3, many similarities can be drawn between differential protein expression in ocular allergy and in keratoconus. Namely, there is an apparent overlap of differentially expressed prolactin-induced protein (PIP), lipocalin-1, lysozyme C, zinc alpha 2-glycoprotein and serum albumin between keratoconus and ocular allergy sufferers [21,47,48,50,51,52,53,54,55]. This is perhaps due to eye-rubbing and inflammatory responses occurring in both conditions.

There does not appear to be much consistency in terms of increased or decreased expression of proteins in ocular allergy sufferers compared to healthy subjects. This is perhaps because current ocular allergy tear proteomics research is limited as the field is still relatively new. Baseline protein characterisations have only just begun to be developed with a consistent methodology and significant sample size. Lack of comparative values and standardised techniques reduce the validity of findings pertaining to specific ocular surface conditions, and thus additional healthy participant studies must be carried out prior to commencing ocular allergy research.

Three of the proteins shown in Figure 3 to overlap in in ocular allergy and keratoconus are known primary protein constituents of healthy human tears—serum albumin, lipocalin-1, IgA and lysozyme C [23]. Lipocalins alone make up approximately 33% of human tear proteins [58,59]. Changes in concentrations of these proteins occurring in both keratoconus and ocular allergy sufferers may indicate roles in immunity, host defence, inflammation, hydration and lubrication on the ocular surface; and the subsequent involvement of eye-rubbing and mechanical stress [60,61,62,63,64,65]. Albumin is secreted by ocular surface conjunctival epithelial cells in response to wounding and irritation, easing symptoms by binding and delivering lubricating lipids to sites of irritation [63]. Lipocalin-1 has also been shown to bind to and regulate lipid distribution on the ocular surface [65]. Prolactin-induced protein (PIP) plays a role in immune modulation and host defence, while zinc alpha 2-glycoprotein (ZAG) function has not yet been characterised (although implicated to be immune-related) [61,64]. Lysozyme C has been affiliated with antimicrobial activity and host defence in the presence of lactoferrin, as well as IgA [60,62]. Due to the commonality of lysozyme C, lactoferrin-1 and IgA and the unknown direction of regulation occurring in either keratoconus or ocular allergy, it is a far more viable suggestion to look at ocular allergy-specific proteins for potential biomarker identification.

Limited available studies in ocular allergy have identified a number of potential diagnostic biomarkers and drug targets in human tears. Proteins such as lactoferrin and IgA were reportedly increased in ocular allergy [40], as were eosinophil cationic protein and eosinophil neurotoxin [3,57]. Combined with the increased concentration of eosinophil major basic protein in ocular allergy, the collective roles appear to be immunomodulatory and antimicrobial in nature [3,57,66,67,68]. The direct functions of these proteins have not yet been fully characterised or compared to clinical measures of symptoms or signs on the ocular surface. A paper by Woerly et al., however, suggested a protein interaction pathway in response to pathogens whereby eosinophils are stimulated by IgA or eotaxin-1 to release eosinophil cationic protein and eosinophil neurotoxin [69]. This interaction would have a net response of increased inflammation at local allergy sites [69]. Eotaxin-1, IgA, eosinophil cationic protein and eosinophil neurotoxin have all been reportedly increased in ocular allergy [3,40,57,70]. 

The antigen–antibody complex forms by binding of allergens to IgE on mast cell surfaces, which triggers mast cell activation and then tryptase is released via degranulation [71]. A 2018 paper investigating a wide array of protein constituents reported increased concentrations of beta-2 microglobulin, lipocalin-1, ZAG, PIP and secretoglobin family 1D member 1 in ocular allergy sufferers compared to healthy controls [51]. In contrast, this same study reported that zymogen granule protein 16 homolog B concentrations had decreased alongside Deleted in Malignant Brain Tumours-1 (DMBT-1) protein and the Ig alpha-1 chain C region in ocular allergy sufferers compared to healthy controls [51]. More recently, a study by Neil et al. in 2020 on ocular allergy sufferers reported that concentrations of Ig light chains, IgG and lactoferrin increased, while ZAG, lysozyme C and lipocalin-1 concentrations all decreased in allergy participants compared to healthy controls [53]. Alpha-defensin, serum albumin, Ig gamma-2 heavy chain C region and leukocyte elastase inhibitor were reportedly also upregulated in ocular allergy patient tears [55,56,70]. While not yet fully characterised in ocular allergy, general functions of these proteins are recorded in Table 1. More research (such as cell culture models) must be done to assess the roles of potential biomarker proteins on the ocular surface in ocular allergy in order to determine how legitimate they may be as candidates for drug targeting and diagnostic testing. Additionally, some studies reported conflicting data pertaining to an increase or decrease in the expression of proteins such as lipocalin-1 and ZAG [51,53]. This may be due to a difference in analysis technique or individual demographics such as age, sex and allergen exposure due to location.

Other potential contributing factors to protein expression inconsistencies between ocular allergy studies on ocular allergy sufferers compared to healthy controls shown in Table 1 may include variations in sample collection method, sample size or accuracy/sensitivity of equipment and techniques. Without a somewhat standardised method, results from multiple studies cannot be compared quantitatively. Instead, general trends may be observed. As this field expands, more and more studies into the healthy tear proteome are emerging. By developing an accurate and consistent comparative model of proteins in healthy human tears, studies looking at specific disorders or conditions of the ocular surface will have a better methodological foundation to build upon. By first developing an accepted normative database of protein contents and concentrations in healthy adults, a method for characterising similar biosignatures for ocular allergy sufferers becomes more feasible. Large-scale and longitudinal studies are most appropriate for creating clinically applicable models of ocular allergy.

Other allergy-associated mediators are granule molecules released by mast cells that directly stimulate inflammation, redness and irritation on the ocular surface [79,80,81]. These mediators include histamine, prostaglandin, tryptase and leukotrienes [79,80,81]. Histamine is responsible for most late-phase allergy symptoms such as redness and inflammation [79], prostaglandins and leukotrienes cause vasodilation and subsequent redness and conjunctival secretions, while also attracting other immune cells to the site [80,81]. The release of these proteins is directly linked to an allergy flare-up, making them an ideal target for anti-allergy medications. A number of antihistamine drugs are currently available over-the-counter in tablet and eye-drop form. This is a management option only and cannot be necessarily used as a preventative measure. Modified proteins, such as mucins (MUC), are also triggered during ocular allergy flare-ups and are yet to be investigated as potential biomarkers. 

Mucins are high molecular weight, heavily glycosylated proteins that are the major macromolecular constituents of the mucus layer of all the major tracts of the human body, including the ocular surface [82]. Mucin production provides lubrication to the conjunctival surface, along with providing a barrier to invading allergens. Mucins are characterised by the presence of extensive *O*-glycosylation. This mesh of *O*-glycoproteins not only protect mucins from proteolytic enzymes, but are also responsible for their rigid, extended conformation [83]. As these carbohydrate moieties constitute about 50–90% of the total mucin mass, it is not surprising that the glycosylation pattern of mucins is critical for their biological function, specifically in the eye. 

Mucins are classified into two categories, based on their location relative to the cell surface—secreted mucins are entirely extracellular as they are secreted by mucus-producing secretory cells and cell-surface (transmembrane, membrane-tethered and/or cell-associated) mucins that are anchored to the cell via a transmembrane domain [82,84,85,86]. MUC1 is a cell-associated mucin that has been studied in dry eye patients. An isoform of MUC1, called MUC1/A, has a lower expression in dry eye patients [82]. This has been attributed to the longer length of this isoform (compared to other isoforms), which provides better lubrication due to higher glycosylation. MUC5AC is a secreted mucin and studies report a reduced secretion of this mucin in patients with dry eye and atopic keratoconjunctivitis [87]. Mucin production is triggered during chronic inflammation and allergic responses to protect the ocular surface by removing allergens from the tear film. Thus, dysregulated mucin production contributes to ocular pathology and has the potential to serve as a biomarker for ocular diseases, including allergy.

## 5. Impact of External Factors on Protein Biosignatures

The normal regulatory tear film in healthy subjects may be affected by individual characteristics such as age and sex. Sex-linked differences in ocular surface composition have not been well documented in ocular allergy, however, have been noted in healthy participants [88]. Sex-associated differences in protein profiles in healthy individuals were addressed in a paper by Versura et al. in 2017 using the AET instrument [88]. Versura et al. noted an increase of lacritin—a tear stimulant and cell survival protein—in males, despite this protein being reportedly unchanged by Seifert et al. [88,89,90]. Sex was not found to modify protein expression in human tears by Nattinen et al. [91]. 

Age has been shown to be an extraneous variable in protein expression on the ocular surface; it has been previously reported that the protein eotaxin increases with age [92]. Expression of other proteins such as lactoferrin were shown to decrease with age, while no significant differences in lacritin have been found [89,93]. Albumin and lipocalin were reportedly also positively correlated with age in a study from 2019, as well as several other regulatory tear proteins not affected by ocular allergy [91]. More research is needed to discern the true impacts of age and sex on the ocular surface proteome of ocular allergy sufferers, as well as other cellular constituents such as mucins. This research would thereby lead the way in the development of new treatment and diagnostic applications using ocular allergy biomarkers.

## 6. Applications of Biomarkers in Prevention and Treatment Strategies

Biomarkers such as proteins and mucins expressed on the ocular surface can be used to update modern therapeutics for ocular allergy. Characterisation of protein and mucin biomarkers of ocular allergy may allow for clinical diagnosis of ‘at-risk’ patients and thus the implementation of earlier intervention strategies. Early intervention will lead to reduced symptoms of itchiness and thus less frequent rubbing of eyes, in turn protecting the cornea from long-term irreversible damage. Current therapeutics for ocular allergy include antihistamines, eosinophil inhibitors, mast-cell stabilisers and nasal corticosteroids for those individuals who seek symptomatic relief in a timely manner. However, those who avoid seeking medical attention may pursue instant relief, alleviating the most common symptom of itch by rubbing their eyes. The initial relief caused by eye rubbing may delay treatment even further, however, as it may lead to increased mechanical stress, and therefore upregulated reactionary protein expression. This perpetuates the symptom cycle on the ocular surface. The true impacts of eye-rubbing on the ocular surface have not yet been researched at the molecular, genetic or cellular level, despite being linked to cornea-altering conditions such as keratoconus [5]. Allergy sufferers may also opt for over-the-counter (OTC) eye drops to relieve the symptoms of ocular allergy. Potential drug targets not contingent on mast cell activity may be highlighted by the results of such research. This would mean that preventative eye-drops antagonising specific proteins could be developed, suppressing the onset of ocular allergy symptom expression and thus reducing the impact of downstream effects such as inflammation on the ocular surface. 

Emerging sales trends of OTC pharmaceutical drugs used to symptomatically treat allergy expose a pattern of unmonitored self-treatment of common allergic conditions such as allergic rhinitis (hay fever) and ocular allergy—thus presenting an attempt by patients to take control and improve upon a reduced quality of life with OTC drugs. Australian cumulative wholesale costs of OTC anti-allergic drugs has more than doubled from 1991 to 2010, showing an increase from $108 million annually to $226 million [94]. These OTC drugs include eye drops, nasal corticosteroids and orally administered drugs. Statistics also show that roughly 56.1% of patients with ocular allergy self-medicate [95]. This trend is indicative of an overwhelming number of potentially unmonitored ocular allergy sufferers not receiving appropriate and personalised treatment. Repeated use of OTC treatments is most likely due to patients not seeking follow-up treatments after being recommended OTC medications, instead opting to repurchase drugs as symptoms flare-up, without seeking further advice. 

Additionally, patients may be over-exposing their eyes to treatments or preservatives found in some of these eye drops that could be harmful if used in excess or not according to directions. It is thereby pertinent that large-scale proteomics studies of human tears be conducted to provide a comparative standard for investigating the potential irritation caused by unmonitored treatments. Biomarker research may also characterise new drug targets that directly implicate ocular allergy biopathways, thus preventing symptom presentation on the ocular surface altogether.

## 7. Conclusions

A combination of clinical screening measures such as questionnaires, physical ocular surface assessments and biochemical tests would be the most effective way to treat the individual. Underutilised and post-symptomatic diagnostic methods for ocular allergy may lead to treatments being implemented too late, therefore greatly impacting the daily quality of life of sufferers. Early detection using biochemical and clinical methods in conjunction would therefore lead to fewer downstream adverse effects of eye-rubbing on the cornea. Symptoms may gradually worsen without appropriate management, as research has shown that OTC medications may cause patients to develop an increasing tolerance that decreases drug efficacy in the long-term [89]. In order to reduce the impact that irritated and itchy eyes have on a patient, specific diagnostic measures must be developed to ensure high-quality results. Having a complete, sophisticated strategy to diagnose ocular allergy, personal triggers and level of sensitivity, as well as the most promising treatment/management options, would significantly reduce the burden of disease of ocular allergy globally. Protein biomarker research conducted on non-invasively collected tear samples may just be key.

## Figures and Tables

**Table 1:** Potential ocular allergy biomarkers identified by previous studies and their associated functions.

Ocular Allergy Protein Biomarker	Function	Change in Concentration (Ocular Allergy versus Healthy Controls)	Analytical Technique
Alpha defensin	Defensins have been shown to be antimicrobial and are able to speed up epithelial tissue healing [42]	Increased [56]	ELISA [56]
Beta-2 microglobulin	Produced by T and B cells, beta-2 microglobulin forms part of the HLA ^†^ class I molecule and has been linked to a number of inflammatory disorders [51,72]	Increased [51]	Mass spectrometry [51]
Deleted in malignant brain tumours 1 protein	Contributes to innate immune reaction control [73]	Decreased [51]	Mass spectrometry [51]
Eosinophil cationic protein	Mast cell degranulation and neutrophil activation [66,68]	Increased [3,57]	Radioimmunoassay [3] Enzyme-linked immunosorbent assay (ELISA) [57]
Eosinophil major basic protein	Mast cell degranulation and immunoregulatory roles [68]	Increased [3,57,66,67,68]	Radioimmunoassay [3] Enzyme-linked immunosorbent assay (ELISA) [57] Concentration-dependent chemiluminescence assay [66] Radioimmunoassay [67]
Eosinophil neurotoxin	Potential inflammatory role [67]	Increased [3,57]	Radioimmunoassay [3] Enzyme-linked immunosorbent assay (ELISA) [57]
Ig ^‡^ light chains	Building block of various immunoglobulins, suggests an increase in Ig ^‡^ A or Ig ^‡^ E production in response to allergy [53,69,74]	Decreased [51] Increased [53]	Mass spectrometry [51] AET [53]
Ig ^‡^ G Ig ^‡^ gamma-2 heavy chain C region	Primarily involved in homeostasis and protection [75]	Increased [51]	Mass spectrometry [51]
IgA	Antimicrobial activity and host defence [62]	Increased [40]	AET [40]
Lactoferrin	Immunomodulatory and antimicrobial when coupled with lysozyme C [60]	Increased [40,53]	AET [40,53]
Leukocyte elastase inhibitor	Anti-apoptotic effects when associated with DNase II [76]. Controls effects of proteinase 3, an enzyme that triggers cell signalling and inflammation [76,77]	Increased [55]	Sodium dodecyl sulfate polyacrylamide gel electrophoresis and Western blot [55]
Lipocalin-1	Binds to and regulates lipid distribution on the ocular surface [65]	Increased [51] Decreased [53]	Mass spectrometry [51] AET [53]
Lysozyme C	Antimicrobial activity and host defence [60]	Decreased [53]	AET [53]
Prolactin-Induced Protein	Plays a role in immune modulation and host defence [61]	Increased [51]	Mass spectrometry [51]
Secretoglobulin Family 1D member 1	Secretoglobins are produced by the lacrimal gland of the eye and are involved in binding lipids to help build and maintain the lipid layer of the tear film [43]	Increased [51]	Mass spectrometry [51]
ZAG	Not yet been characterised (though has been suggested to be immune-related) [64]	Increased [51] Decreased [53]	Mass spectrometry [51] AET [53]
Zymogen granule protein 16 homolog B	Unknown, though potentially plays a protective role on the ocular surface [78]	Decreased [51]	Mass spectrometry [51]

^†^ HLA—human leukocyte antigen complex, ^‡^ Ig—immunoglobulin.

## Data Availability

Not applicable.
